# Comparative MRI analysis of the forebrain of three sauropsida models

**DOI:** 10.1007/s00429-024-02788-2

**Published:** 2024-03-28

**Authors:** S Jiménez, I Santos-Álvarez, E Fernández-Valle, D Castejón, P Villa-Valverde, C Rojo-Salvador, P Pérez-Lloret, M. J. Ruiz-Fernández, S. Ariza-Pastrana, R. Martín-Orti, Juncal González-Soriano, Nerea Moreno

**Affiliations:** 1 Achucarro Basque Center for Neuroscience, Scientific Park of the University of the Basque Country (UPV/EHU), Leioa, Bilbao, 48940 Spain; 2 Departament Section of Anatomy and Embriology, Faculty of Veterinary, Complutense University, Avenida Puerta de Hierro s/n, Madrid, 28040 Spain; 3 ICTS Bioimagen Complutense, Complutense University, Paseo de Juan XXIII 1, Madrid, 28040 Spain; 4 Palmitos Park Canarias, Barranco de los Palmitos, s/n, Maspalomas, Las Palmas, 35109 Spain; 5 Department of Cell Biology, Faculty of Biological Sciences, Complutense University, Avenida José Antonio Nováis 12, Madrid, 28040 Spain

**Keywords:** Reptiles, *Trachemys*, Pogona, Python, Magnetic resonance imaging, Brain, Ventricles, Atlas

## Abstract

The study of the brain by magnetic resonance imaging (MRI) allows to obtain detailed anatomical images, useful to describe specific encephalic structures and to analyze possible variabilities. It is widely used in clinical practice and is becoming increasingly used in veterinary medicine, even in exotic animals; however, despite its potential, its use in comparative neuroanatomy studies is still incipient. It is a technology that in recent years has significantly improved anatomical resolution, together with the fact that it is non-invasive and allows for systematic comparative analysis. All this makes it particularly interesting and useful in evolutionary neuroscience studies, since it allows for the analysis and comparison of brains of rare or otherwise inaccessible species. In the present study, we have analyzed the prosencephalon of three representative sauropsid species, the turtle *Trachemys scripta* (order Testudine), the lizard *Pogona vitticeps* (order Squamata) and the snake *Python regius* (order Squamata) by MRI. In addition, we used MRI sections to analyze the total brain volume and ventricular system of these species, employing volumetric and chemometric analyses together. The raw MRI data of the sauropsida models analyzed in the present study are available for viewing and downloading and have allowed us to produce an atlas of the forebrain of each of the species analyzed, with the main brain regions. In addition, our volumetric data showed that the three groups presented clear differences in terms of total and ventricular brain volumes, particularly the turtles, which in all cases presented distinctive characteristics compared to the lizards and snakes.

**Supplementary Information:**

The online version contains supplementary material available at https://doi.org/10.1007/s00429-024-02788-2.

## Introduction

Reptiles are within the largest group of those considered exotic animals. There are more than 10.000 living species, are classified into four orders: Crocodilia (crocodiles and alligators), Sphenodontia or tuataras, Squamata (lizards and snakes) and Testudines turtles and tortoises (Doneley 2017). Through genomic data, reptiles can be grouped into two large groups: Squamata (lizards and snakes) and Archosauria (dinosaurs, modern birds and crocodiles), the group to which turtles are evolutionarily closest (Striedter, 2005; Field et al. 2014; Naumann et al. 2015).

Research in comparative neuroanatomy has revealed a fundamental organization of the vertebrate brain. Reptiles, with their great diversity and interesting phylogenetic position with respect to mammals, are in fact a key model for understanding the anatomy-function relationship of vertebrate brain circuits (Nomura et al. 2013; Kabelik and Hofmann 2018; Laurent 2020). The principal idea is that their brains have, in general, a simple organization, but the reptilian forebrain has many similarities to that of mammals and birds [see ten (ten Donkelaar 1998)].

Magnetic resonance imaging (MRI) is a non-invasive technology that allows an accurate anatomical assignment of a concrete region, or even to analyze possible interhemispheric and individual variabilities by means of tomography. In terms of research, there are interesting papers that focus on different species and demonstrate the usefulness of this technique (Kabli et al. 2006; Dorr et al. 2008; Poirier et al. 2008; Yopak and Frank 2009; Watanabe et al. 2010; Ullmann et al. 2010b; Ullmann et al. 2010a; Lauridsen et al. 2011; Ullmann et al. 2012, 2013a, 2014a, 2015a, b; Derix et al. 2014; Schmidt and Payne 2015; Głodek et al. 2016; Ruiz-Fernández et al. 2020; González et al. 2023). Quantitative data obtained from the segmentation of the different selected regions have an added value together with the application of chemometric analysis techniques that contribute to the classification and identification of these cerebral zones, especially useful in comparative neuroanatomical studies (Corfield et al. 2008; Oelschläger et al. 2008; Yopak and Frank 2009; Tullo et al. 2019).

MRI has also proven its usefulness to study the nervous system of different reptile species (Jirak and Janacek 2017; Anderson et al. 2000; Mathes et al. 2017; Libourel et al. 2018; Schrenk et al. 2022), which in some cases, has resulted in a number of precise anatomical maps and brain atlases (Hoops et al. 2017, 2018, 2021; Behroozi et al. 2017; Billings et al. 2020; Foss et al. 2022). Nevertheless, while comparative studies of different reptile orders have utilized histological classical techniques with higher cellular-level resolution [reviewed in (ten Donkelaar 1998)], brain comparative in vivo analysis using MRI is limited. However, this provides a unique opportunity for comparative neuroanatomical studies, avoiding potential alterations of the tissue processing, particularly when analyzing structures such as the ventricles, the choroid plexus or the simple general anatomy of the reptilian brain. In addition, the non-invasive nature of MRI opens the door to a wider range of species that might otherwise be challenging to study, undoubtedly improving our understanding of the evolution of amniotes and the differences between different groups of reptiles (Nomura et al. 2014; Laurent 2020). Therefore, the MRI can be a useful and key complementary tool in neuroanatomical studies.

The main goal of the present study was to use MRI to segment and identify different prosencephalic structures in three representative sauropod species, the turtle *Trachemys scripta* (order Testudine), the lizard *Pogona vitticep*s (order Squamata) and the snake *Python regius* (order Squamata). In addition, this technique was used to examine the total brain volume and the ventricular system of these species, by the use of volumetric and chemometric analyses Our results allows the identification of the different regions and fiber tracts of the forebrain. However, and due to technical limitations, it was not possible to determine, for example, the organisation into layers or the presence of small nuclear clusters.

## Materials and methods

**Fig. 1:**
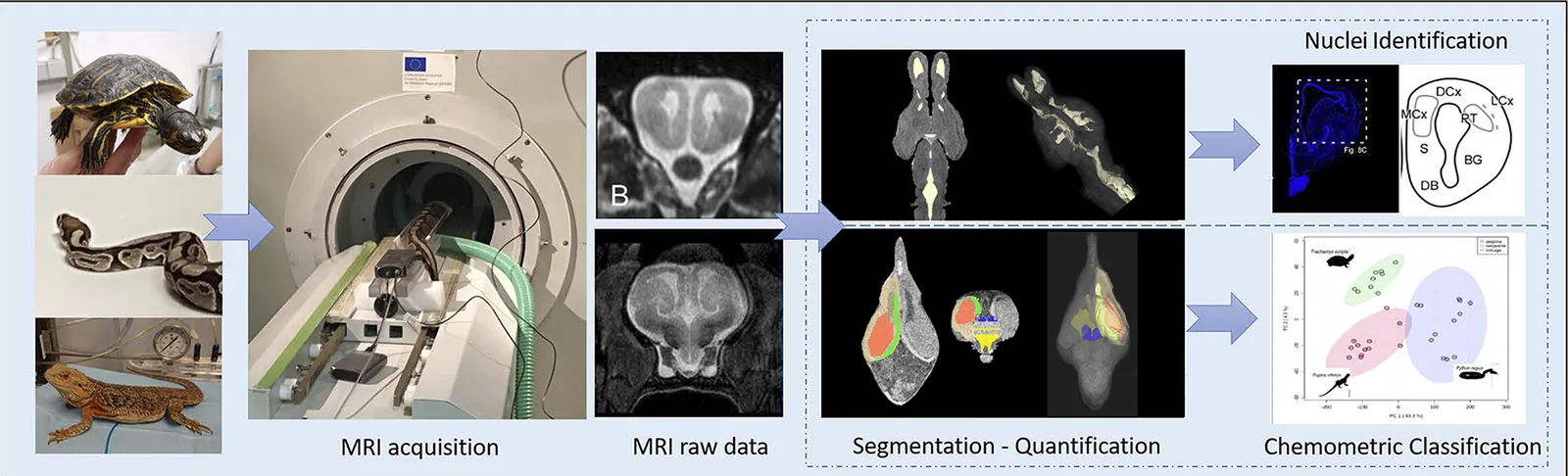
Schematic process of turtle, snake and lizard brain segmentation and classification by magnetic resonance imaging and histology

Figure 1 summarizes the workflow described in the materials and methods from the acquisition of the MRI data to the nuclei identification and chemometric classification of the three species.

### Animals

For the present study we used the turtle *Trachemys scripta* (*n* = 8 and weight between 1000 and 1300 g), the lizard *Pogona vitticeps* (*n* = 11 and weight between 250 and 400 g) and the snake *Python regius* (*n* = 13 and weight between 800 and 900 g). All individuals used, in all three species, were adults, homogeneous in size, and of both sexes. The original research reported herein was performed according to the regulations and laws established by European Union (2010/63/EU) and Spain (Royal Decree 118/2021) for care and handling of animals in research and after approval from the Complutense University to conduct the described experiments.

### Magnetic resonance imaging (MRI) studies in vivo

MRI studies were performed at BioImaC (ICTS BioImagen Complutense, Madrid, Spain), node of the ICTS ReDIB (https://www.redib.net/). Due to the animal sizes in vivo MRI studies were carried out on a Bruker Biospec 47/40 (Bruker Biospin GmbH, Ettligen, Germany) operating a 4.7 Tesla and with a bore size of 40 cm. This system was used due to: (1) the magnetic field strength that allows obtaining a good signal to noise ratio (S/N) in a short experimental time; (2) the maximum size of the bore that allows imaging animals as large as turtles; (3) the different gradient systems and radiofrequency coils that allow imaging animals as different in size and shape as those used in this study.

Animals were anesthetized previously to the MRI studies and were then maintained using a mixture of isofluorane in oxygen throughout the whole experiment. For the initial anesthesia of the turtles, a dose of 10 mg/kg of alfaxalone (Alfaxán, Altres lab., code 520,029) was used intravenously in the dorsal venous sinus or jugular vein. Lizards were anesthetized using an intramuscular injection in the hind limbs with a mixture of ketamine (20 mg/kg) (Ketaset, Ecuphar lab., Albet code. 9,400,089) and butorphanol (1.5 mg/kg) (Butomidor, Karizoo lab., code 01520064). Using the same mixture, snakes were anesthetized intramuscularly with and injection in the dorsal muscles of the second third of the snake’s total length. As we have mentioned before, the anesthesia (a mixture of isoflurane in oxygen) was maintained along the MRI experiment by using a facemask. In all cases, the doses were always dependent on the depth of the anesthesia. The respiration and the temperature of the animals were controlled and monitored by using the SA 1030 system (Small Animal Instruments, Inc., Nueva York, USA). The total acquisition time never exceed 2 h. At the end of the imaging experiment, animals were recovered by means of different systems to raise the animals’ temperature as, for example, electrical blankets or warm air currents.

The size of the gradient system and radiofrequency coil depended on the size and anatomy of each specie. For *Trachemys scripta*, we used a 26 cm gradient system and a 6 cm shaped surface radiofrequency coil. For *Pogona vitticeps*, the internal diameter of the gradient system was 12 cm whereas the volume radiofrequency coil was 7 cm. Finally, for *Python regius* we used a gradient system of 6 cm and a volume radiofrequency coil of 3.5 cm. In all cases, after some fast images for localizing the brain, a 3D T2-weighted fast spin-echo sequence with a repetition time of 2700 ms and an effective echo time of 95 ms was acquired. Depending on different factors, such as the species and the animal weight, the field of view varied from 30 × 30 × 15 mm^3^ to 30 × 15 × 7.5 mm^3^ and the resolution between 117 × 117 × 117 µm^3^ to 66 × 66 × 66 µm^3^.

### MRI ex vivo

Due to the better contrast between tissues obtained at low magnetic field and the reduced size of the ex vivo samples, a 1-Tesla benchtop MRI scanner [Icon (1T-MRI); Bruker BioSpin GmbH, Ettlingen, Germany] was used for ex vivo studies. The system consists of a 1 T permanent magnet (without extra cooling required for the magnet) with a 6 cm gradient coil that provides a gradient strength of 450 mT/m. and a solenoid RF coil (inner diameter 23 mm and length 25 mm. In the case of those brains used for histological analysis (*n* = 2 in each case, see below), the animals were put under deep anesthesia and after in vivo MRI analysis, they were decapitated and their brains were removed and kept in a fixative solution (4% paraformaldehyde in a 0.1 M phosphate buffer, pH 7.4) until ex vivo MRI study.

For ex vivo MRI acquisition, brains were drained and immersed in a proton-free susceptibility-matching fluid, Fluorinert® FC-40 (Sigma‐Aldrich, Saint Louis, MO, USA). The MRI experiment consisted of three dimensional T2 coronal weighted images (T2WI) used for identification of brain structures. Three-dimensional T2WI were obtained using a rapid acquisition with relaxation enhancement (RARE) technique, with a repetition time (TR) = 2500 s, echo train length = 8, interecho interval = 27 ms (resulting in an effective echo time (TE) = 80 ms), number of averages = 5, field of view (FOV) = 25 × 18 × 14 mm^3^. The acquired matrix size was 250 × 90 × 70 (resolution 0.100 mm x 0.200 mm x 0.200 mm) and the total acquisition time ~ 90 min. Coronal images were reconstructed in axial orientation (isotropic resolution of 0.1 mm^3^). The supplementary material provides details on additional acquired experiments not shown in the text (see supplementary material).

### Histology and imaging

The brains were cryoprotected (30% sucrose in PB for 4–6 h at 4ºC), embedded (7.5% gelatin with 30% sucrose in PB frozen at -80ºC) and cut on a freezing cryostat at 40 μm thickness in the transverse plane. Sections were collected on SuperFrost slides and cover slipped with fluorescence mounting medium, containing 1.5 µg/ml 4’,6-diamidino-2-phenylindole (DAPI) for DNA counterstaining (Santa Cruz; SC-24,941). The sections were analyzed using an Olympus BX51 microscope and were photographed with a digital camera (Olympus DP74). Contrast and brightness were adjusted with Adobe Photoshop CS6 (Adobe Systems, San Jose, CA) and figures and drawings were mounted with Canvas X Draw (ACD Systems, Canada). Using Canvas X Draw, the line drawings in Figs. 3, 4 and 5 were traced following the MR images as a model, in combination with the DAPI images, for the identification of possible boundaries and/or cores. In those regions where the combination of both techniques did not allow the identification of their boundaries, these were not included in the drawings.

### MRI analysis, segmentation and classification

The images for the segmentation of the ventricles and the encephalon were acquired in vivo using a 4.7 Tesla MRI system (explained above). Technically this system allows us to analyze larger live animals during short times with optimum signal to noise ratio. Whereas the images for the segmentation of the nuclei and elaboration of the atlases were acquired using a 1 Tesla MRI system (explained above), since this system allows better contrast ratio, and using ex vivo samples, allowing longer acquisition times.

Each experimental approach was chosen for technical characteristics, although the results with both were comparable. All MRI data was analyzed using ImageJ software (version 1.53 J, Wayne Rasband, NIH, https://imagej.nih.gov/ij/). T2-weighted images were used for their better inter-tissue contrast to identify and segment the main brain nuclei and regions. A three-panel view (coronal, sagittal and transverse orientations) was used to identify the largest number of prosencephalic structures in the MRI sections. Brain segmentation of the sauropsida models was performed manually. To reduce operator-dependent inconsistencies, ventricular segmentation was performed by at least two observers. The ventricular system was divided into lateral ventricles, (aqueduct + III ventricle) and IV ventricle. For the three species, absolute brain volume (mm^3^), total ventricular volumes (mm^3^) and relative ventricular volumes (versus total brain and ventricular volumes) were calculated.

### Statistical analysis

Multivariate Analysis by Principal Component Analysis (PCA) is a statistical method that uses PCA to analyze multiple variables at once. Principal Component Analysis is an unsupervised method used in data analysis and statistics to reduce the dimensionality of a data set by identifying patterns and relationships between the variables. PCA transforms the variables in a dataset into a smaller number of new latent variables called principal components (PCs), which are uncorrelated to one another and account for decreasing proportions of the total variance of the original variables. Each new principal component is a linear combination of the original variables. PCA is an unsupervised method of classification, requiring no training set of data. These principal components are ordered by the amount of variance they explain in the data, with the first component explaining the most variance and each subsequent component explaining less and chosen to be orthogonal to the first PC. The goal of PCA is to explain as much of the variation in the data as possible while reducing the number of variables needed to describe the data. It is widely used in many fields, including metabolomics, image processing, bioinformatics, and finance.

For pattern analysis, brain segmentation described above was used to the analysis. Principal components analysis (PCA) was applied to this data and used no scaling (mean-centered only). Loadings plots from the PC analysis were used to identify pattern. Such analysis was performed with the web-based platform MetaboAnalysis 5.0 (www.metaboanalyst.ca).

## Results

### Anatomical description

**Fig. 2:**
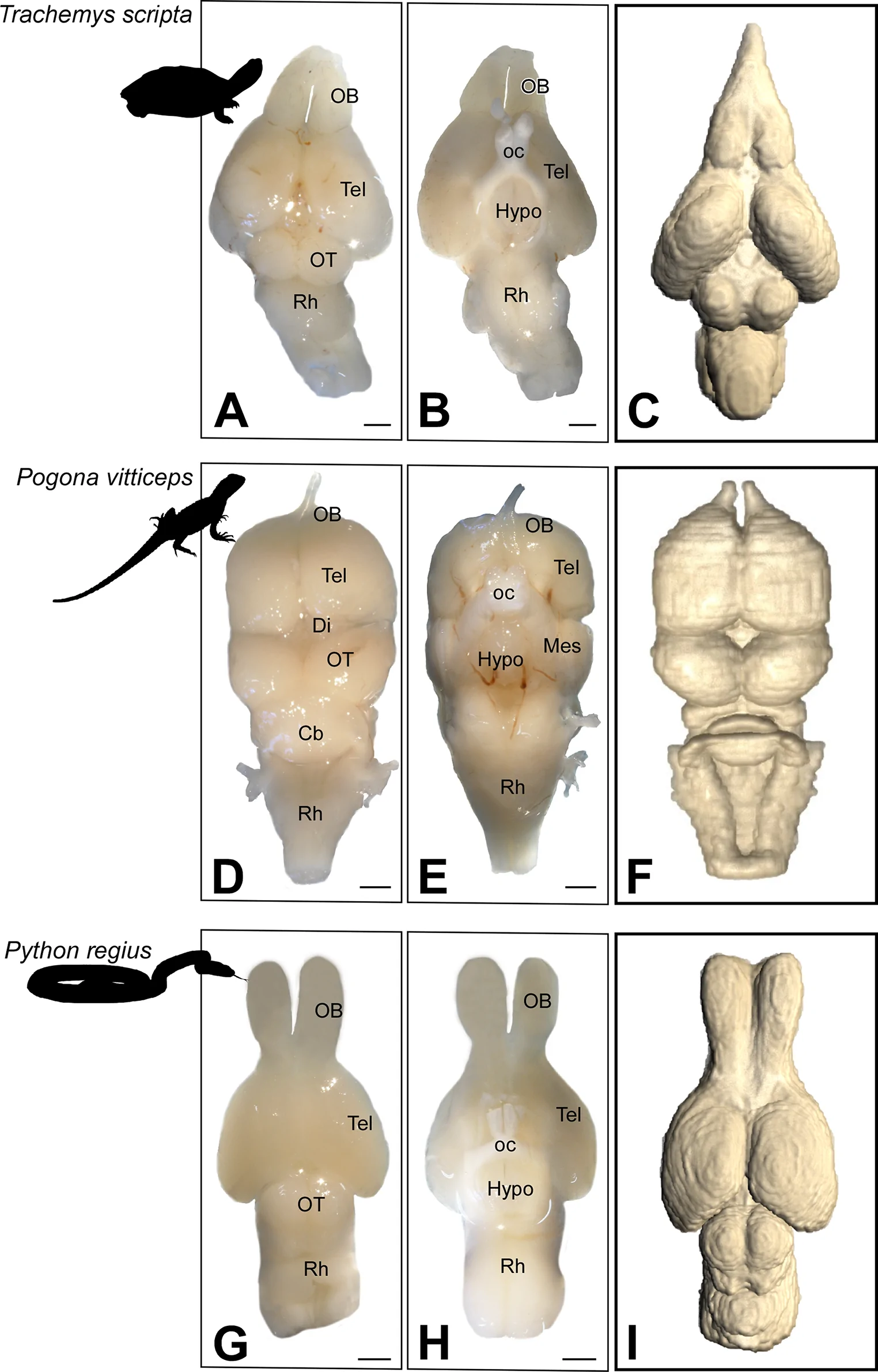
Photos of the brains of *Trachemys scripta* (**A**-**C**), *Pogona vitticeps* (**D**-**F**), and *Python regius* (**G**-**I**) in dorsal (**A**, **D**, **G**) and ventral (**B**, **E**, **H**) views and their 3D reconstructions (**C**, **F**, **I**) based on MR images. The scale bar = 1 mm. See list of abbreviations

**Fig. 3:**
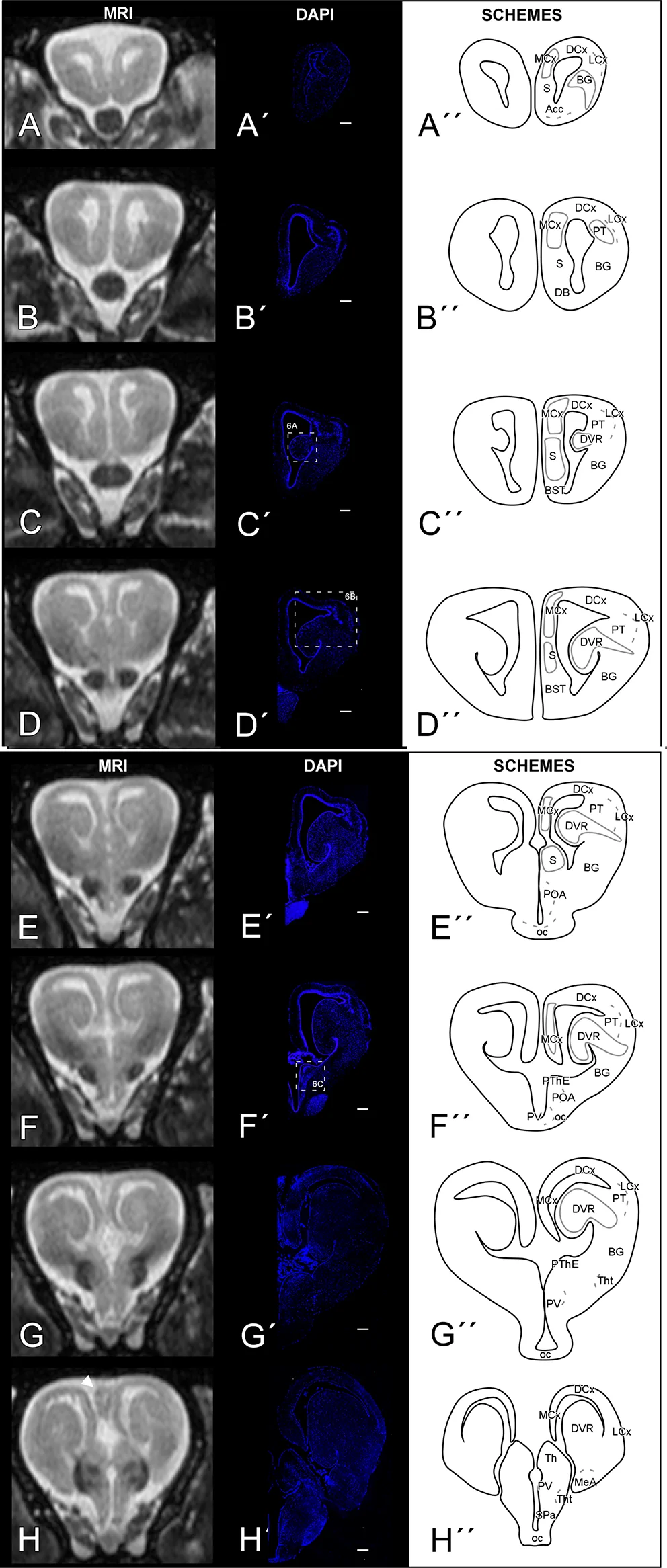
Left column: rostral-caudal coronal sections through the MRI of *Trachemys scripta* brain. Middle column: rostral-caudal coronal histological sections from fluorescent nuclear staining (DAPI). The arrowhead indicates the epiphysis. Right column: schematics of coronal sections indicating the main regions of the forebrain. The scale bar = 500 μm. See list of abbreviations

**Fig. 4:**
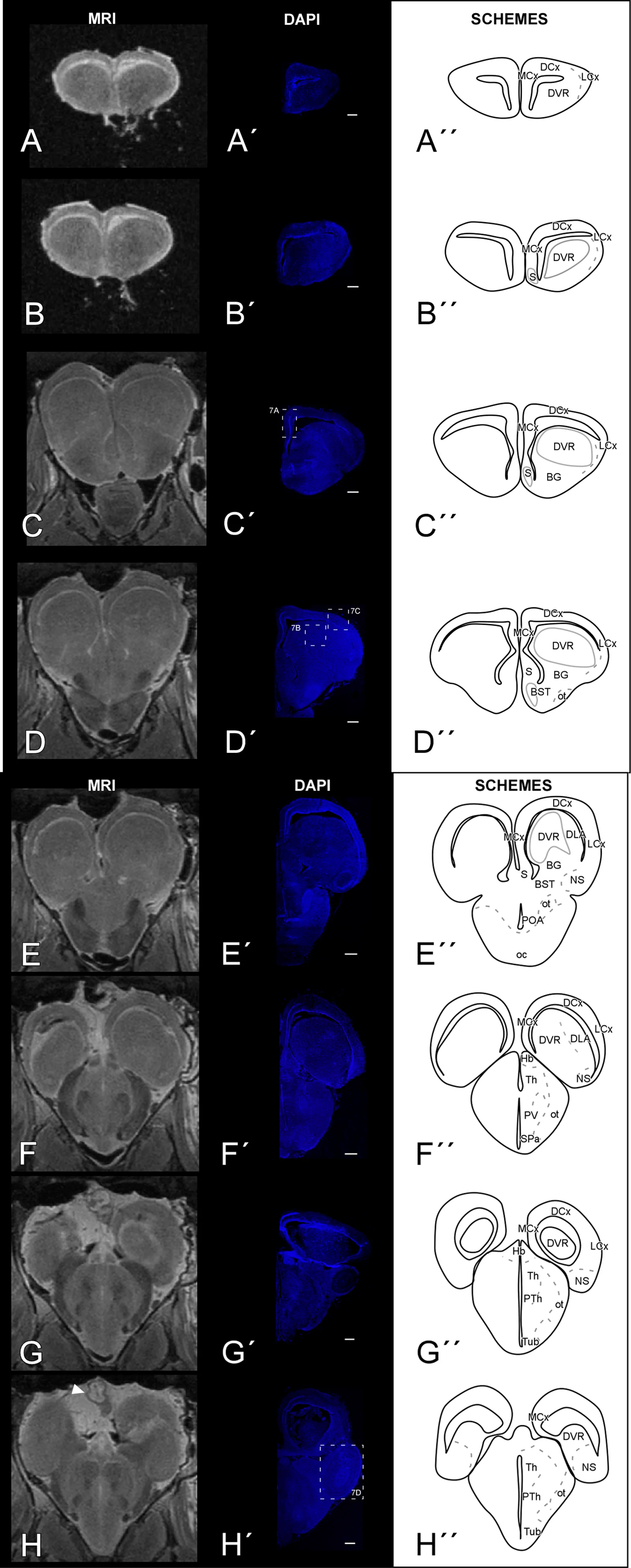
Left column: rostral-caudal coronal sections through the MRI of *Pogona vitticeps* brain. Middle column: rostral-caudal coronal histological sections from fluorescent nuclear staining (DAPI). The arrowhead indicates the epiphysis.Right column: schematics of coronal sections indicating the main regions of the forebrain. The scale bar = 500 μm. See list of abbreviations

**Fig. 5:**
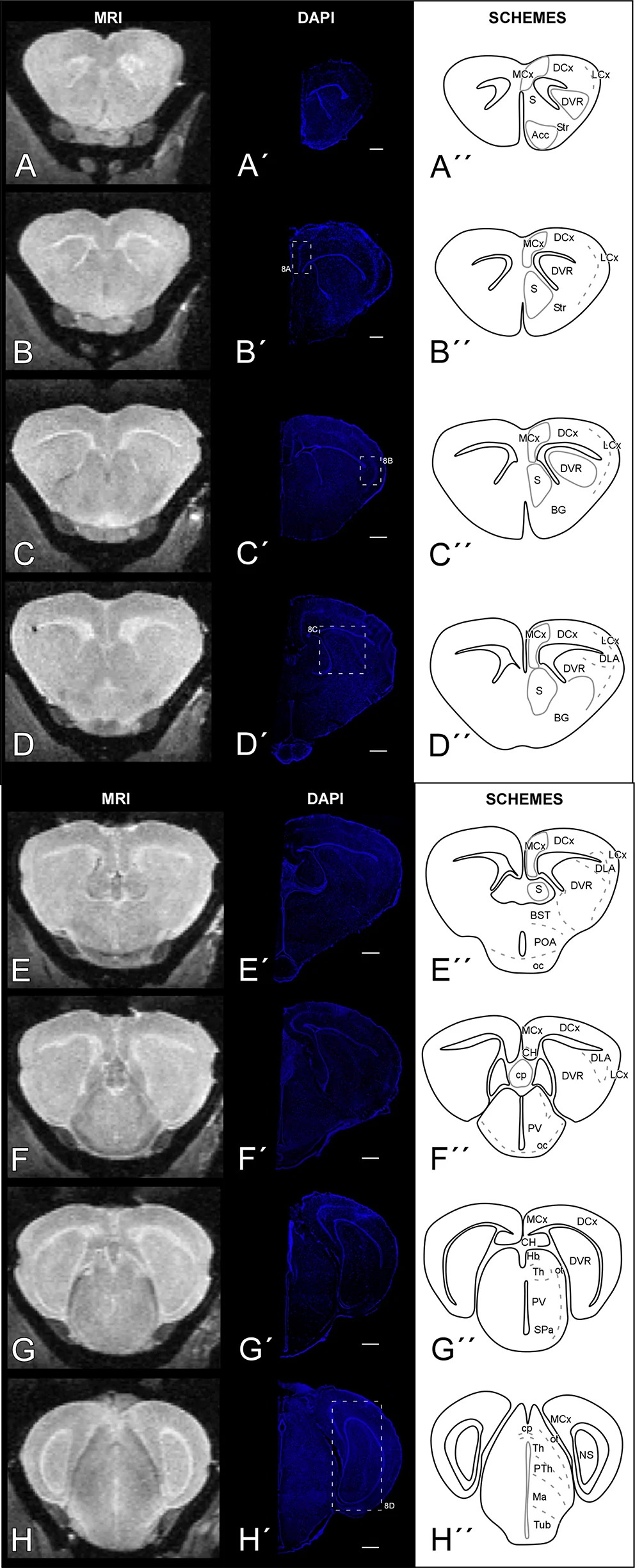
Left column: rostral-caudal coronal sections through the MRI of *Python regius* brain. Middle column: rostral-caudal coronal histological sections from fluorescent nuclear staining (DAPI). Right column: schematics of coronal sections indicating the main regions of the forebrain. The scale bar = 500 μm. See list of abbreviations

This work resulted in a neuroanatomical atlas of three different reptile brains. Thus, in the present study we identified different prosencephalic regions and structures by analyzing the ex vivo brains of the turtle *Trachemys scripta* (Fig. 2A-C), the dragon *Pogona vitticeps* (Fig. 2D-F) and the snake *Python regius* (Fig. 2G-I), by means of MRI and DAPI nuclei staining (Figs. 3, 4 and 5). The nomenclature was taken from previous studies related to the encephalon of these animals (ten Donkelaar 1998).

**Fig. 6:**
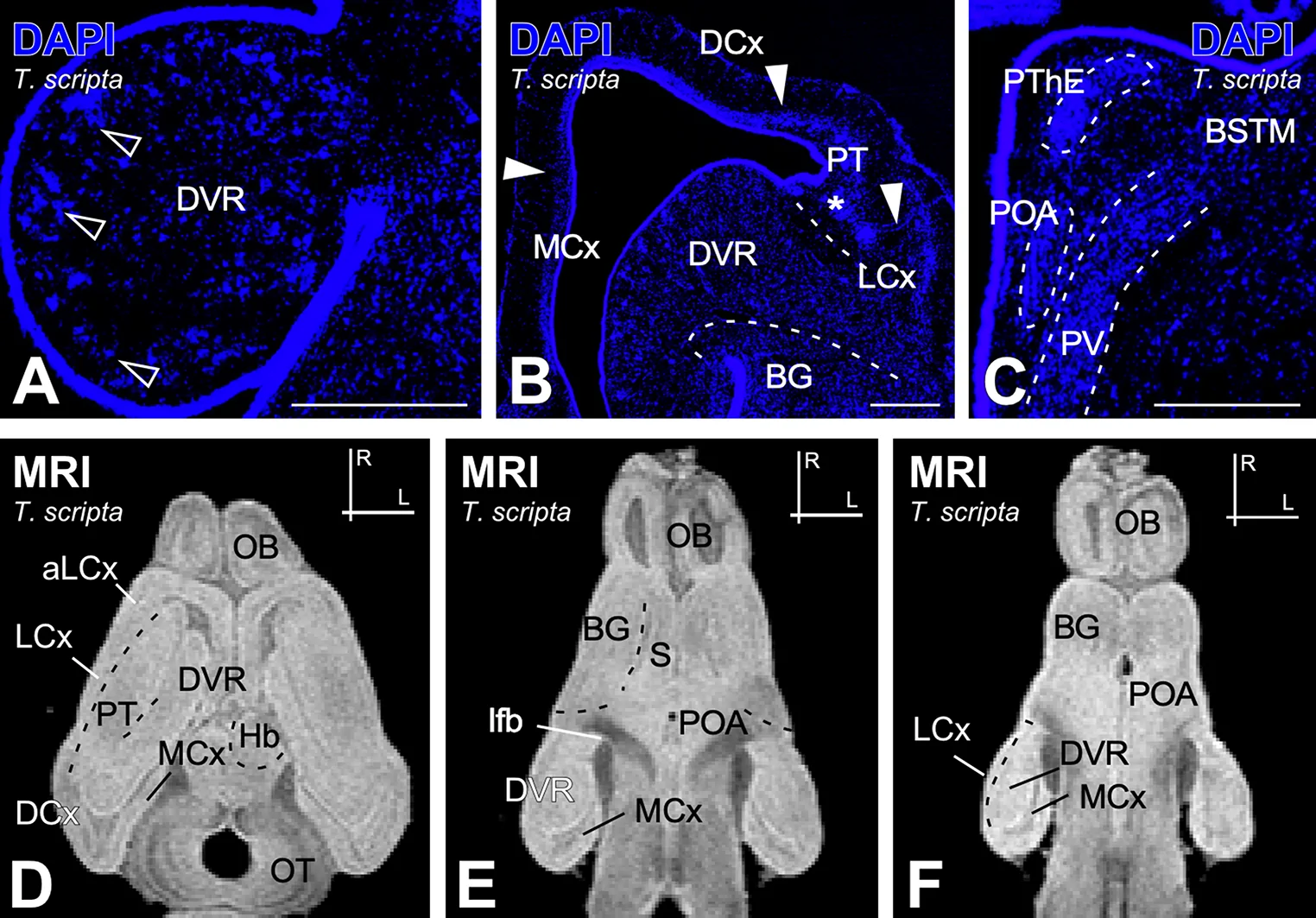
Photomicrographs of transverse sections through the adult telencephalon of *Trachemys scripta* showing nuclear staining by DAPI (**A**-**C**) and horizontal MRI sections (**D**-**F**). Filled arrowheads indicate the layered organization. Empty arrowheads point to cell clusters. In the MRI images the orientation is indicated at the right, the DAPI images (**A**-**C**) are from coronal sections. Scale bar in A, C = 200 μm and B = 500 μm. See the abbreviation list

**Fig. 7:**
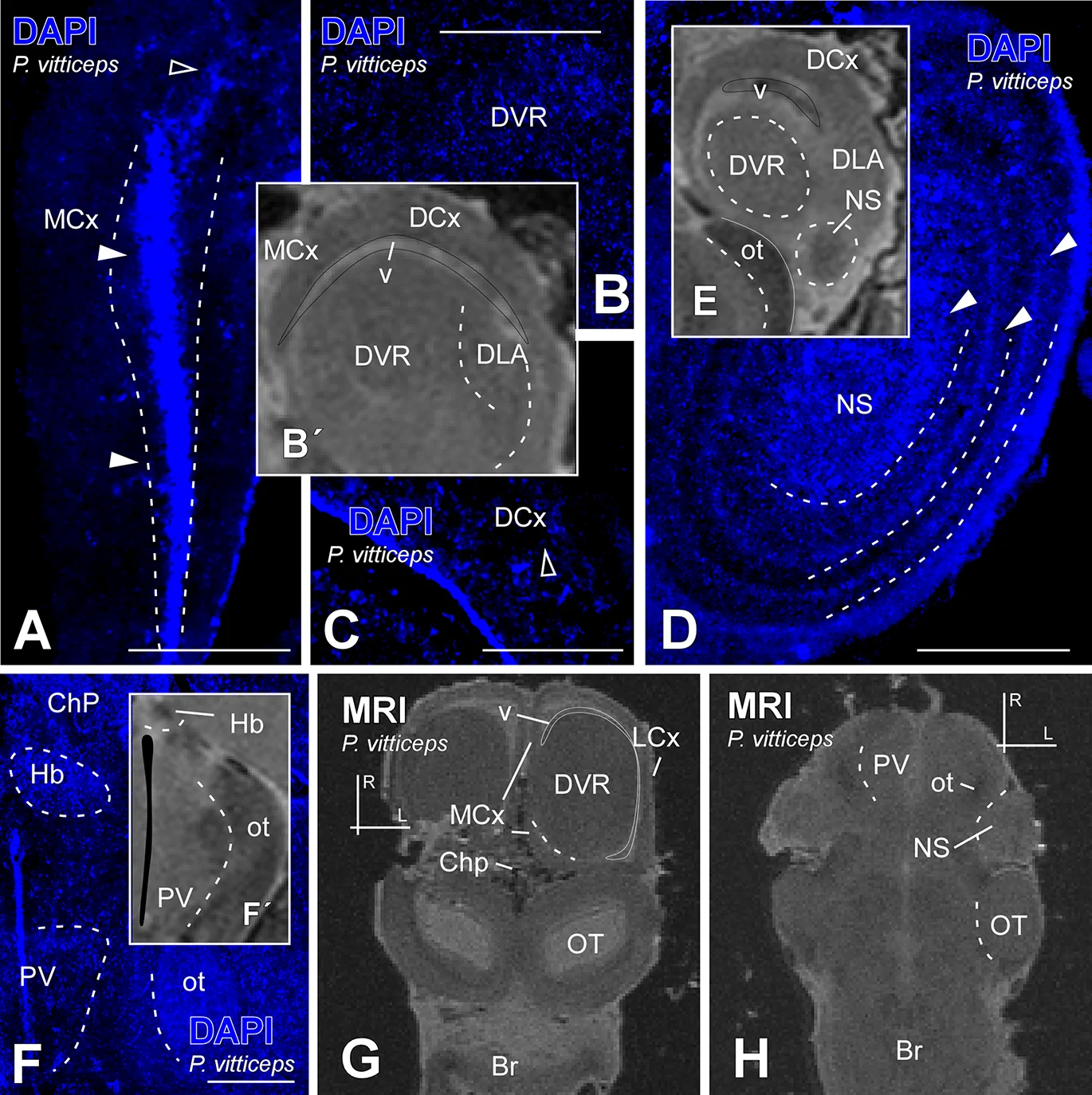
Photomicrographs of transverse sections through the adult telencephalon of *Pogona vitticeps* showing nuclear staining by DAPI (**A**–**F**) and MRI sections (**B**´, **E**, **F**´, **G**, **H**). In the horizontal MRI images the orientation is indicated at the right, the other images, MRI and DAPI, are coronal orientated. Scale bar in A-D = 500 μm and F = 200 μm. See the abbreviation list

**Fig. 8:**
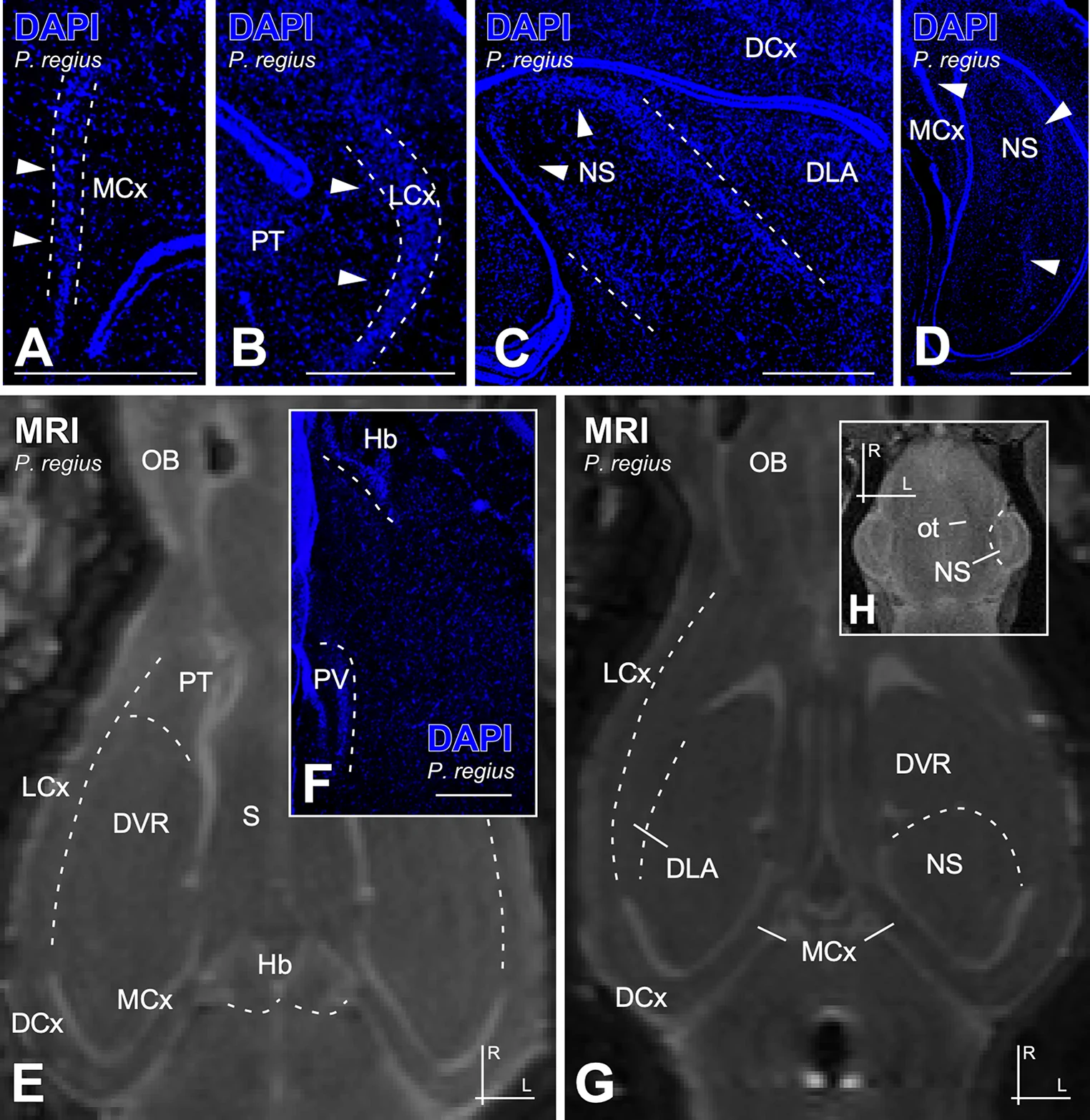
Photomicrographs of transverse sections through the adult telencephalon of *Python regius* showing nuclear staining by DAPI (**A**-**D**, **F**) and horizontal MRI sections (**E**, **G**, **H**). In the MRI images the orientation is indicated at the right (**E**, **G**) and the left (**H**), the DAPI images (**A**-**D**, **F**) are from coronal sections. Scale bar in A-D = 500 μm and F = 200 μm. See the abbreviation list

The anatomical findings are shown in a combination of coronal MRI and DAPI sections, together with the corresponding drawings (Figs. 3, 4 and 5). To verify the cellular organization, specific regions of the forebrain were analyzed and compared at high magnification (Figs. 6, 7 and 8). Subsequent segmentation analyses were performed on in vivo material to avoid possible alterations due to the fixation process, therefore the images were acquired on the 4.7 Tesla scanner as indicated in material and methods. In the case of the images used for the neuroanatomical atlas, they were acquired on fixed material, i.e. ex vivo, as indicated above. No significant differences were found between the two scanners. The MRI raw data of the sauropsida models analyzed in the present study are available for viewing and downloading at the folder MRI DATA OF SAUROPSIDA MODELS (https://drive.google.com/drive/folders/1QsQVL6S6oz_mRXavYt4LpCXtSKJgxbWZ?usp=sharing).

Brain regions that have the highest water content are generally zones with a high concentration of cell bodies and neuropil. On the contrary, fiber tracts, appear hypointense in the MR images due to lower transverse relaxation time T_2_. These differences were more remarkable in the turtle (Fig. 3), in comparison to the other two models.

Our MRI images allowed the identification of different cortical regions in the telencephalon. Thus, the cerebral cortex was divisible in different areas, according to their relative position: the medial, dorsal and lateral cortex. The medial cortex (MCx; Figs. 3, 4 and 5) was located above the septum and showed differences between the different models of our interest. In the case of the turtle, the extension of the medial cortex was clearly greater (Fig. 3), in comparison to the other two models (Figs. 4 and 5). In this region, a common feature of turtle, lizard and snake, that it was not apparent by MRI was the layering arrangement. Thus, one of the major variations between the two methods at this level is the differentiation of cellular organization. In particular, DAPI analysis revealed a large layer of cells surrounded by two adjacent layers of low-cell density (Figs. 6B and D-F and 7A and B´, G; 8 A, D, E, G). In the dorsal cortex (DCx), when using MRI, it was not possible to distinguish a layered structure in none of the three models (see MRI columns in Figs. 3, 4 and 5). In contrast, the DAPI analysis (see DAPI columns in Figs. 3, 4 and 5) showed that there are notable differences in the cellular organization of these layers in the species studied (Figs. 6B and D and 7B´, C, E; 8C, E, G). In turtles, there is a major cellular layer (Fig. 6B) whereas in lizard the cells are layered in clusters (see empty arrowhead in Fig. 7C). In addition, the MRI analysis showed remarkable size differences in this dorsal region between the three models. The lateral cortex (LCx) extends throughout the rostro-caudal extent of the lateral border of the pallium (Figs. 3C, 4D and E and 5E). Like the rest of the cortical structures in the three models, the LCx was anatomically localized by MRI, although its layered organization was only visible by histological analysis (see filled arrowheads in Figs. 6B and 8B). Another structure which was visible in our MRI images and confirmed by means of DAPI, was the pallial thickening (PT). This telencephalic region varied significantly according to the species, being in the turtle where it was most clearly visualized (Fig. 6B, D), in contrast to the other models. In addition, in this dorsolateral region in lizards and snakes a region belonging to the amygdala, the dorsolateral amygdala (DLA), has been described (Lanuza et al. 1998; Lanuza and Halpern 1998), which can be localized in our models (Figs. 4E, 5D-F and 7B’, E; 8C, G). Close to this dorsolateral domain, it was possible to identify the dorsal ventricular ridge (DVR), which was present in the three models (Figs. 3, 4 and 5). At this level, the main technical differences between MRI and DAPI were the possibility to make an identification of the cellular organization. It was necessary to use the contrast of the MRI images in combination with the DAPI sections to observe its cellular organization: in patches in the case of turtles (see empty arrowheads in Fig. 6A and compare to 6D-F) and more uniform in Pogona (Fig. 7B, B´, E, G). At caudal telencephalic levels, another telencephalic structure clearly identified by MRI was the nucleus sphericus (NS), a layered formation within the DVR, which has been described in different reptile models [see (ten Donkelaar 1998)]. It was very visible in lizard (Fig. 7D, E, H) and snake (Fig. 8C, D, G, H).

In the subpallium, the regions present in the three models were analyzed rotrocaudally (Figs. 3, 4 and 5). At rostral levels, the basal ganglia (BG) were identified (Figs. 3A-D, 4A-D and 5A-D). In all three cases, this region, probably due to the greater presence of fibers, shows a higher intensity on MRI images, making it an anatomically easily identifiable area on MRI. However, this imaging technique did not allow to identify the tracts with histological precision, although they must correspond with the described basal ganglia outflow tracts. In the medial territory, the region described as septum (S) was clearly identified in all models (Figs. 3A-D, 4A-D and 5A-D). In this case, no differences in intensity were identified by MRI. However, its identification and comparative analysis were noteworthy, because they highlight the significant difference in size between the three species. It is interesting to point out that in the pogona was comparatively smaller than in the turtle and the snake. The lateral ventricles were hypertense in T2 weighted MRI contrast and therefore easily identifiable. The combination of MRI and DAPI sections enabled the identification of the hypothalamus as well as the presence of large fiber tracts in turtles, lizards, and snakes. While with MRI was impossible to identify the considerable number of nuclei present in this area, DAPI revealed differences in cell types, although did not allow a clear nuclear recognition (Figs. 3, 4 and 5). In this region major fiber tracts can comparatively be identified such as the optic chiasma (oc; Figs. 3E and F, 4E and F and 5E and F), the optic tract (ot; Figs. 4D-H and 5G and H) and the thalamic-hypothamic tract (Tht; Fig. 3G, H). In addition, as described in the case of the lateral ventricles, the third ventricle appears hyperintense on the T2-weighted image, making it easily identifiable.

At diencephalic levels, the prethalamic eminence (PThE) was identified in turtles by MRI (Fig. 3F, G), and confirmed histologically (Fig. 6C). The epiphysis or pineal gland was visible in turtles and lizard (Figs. 3H and 4H), whereas the dorsal diencephalon showed the habenular complex in the three models of our interest (Figs. 3H, 4H and 5G), results confirmed by histology (Figs. 6D and 7F-F´; 8E, F). It is interesting to remark that, in the rest of the diencephalon, especially the structures of thalamic origin - which are very bulky in comparison to each other - were correctly visualized by MRI (Figs. 3H, 4F-H and 5G and H).

### Volumetric analysis

**Fig. 9:**
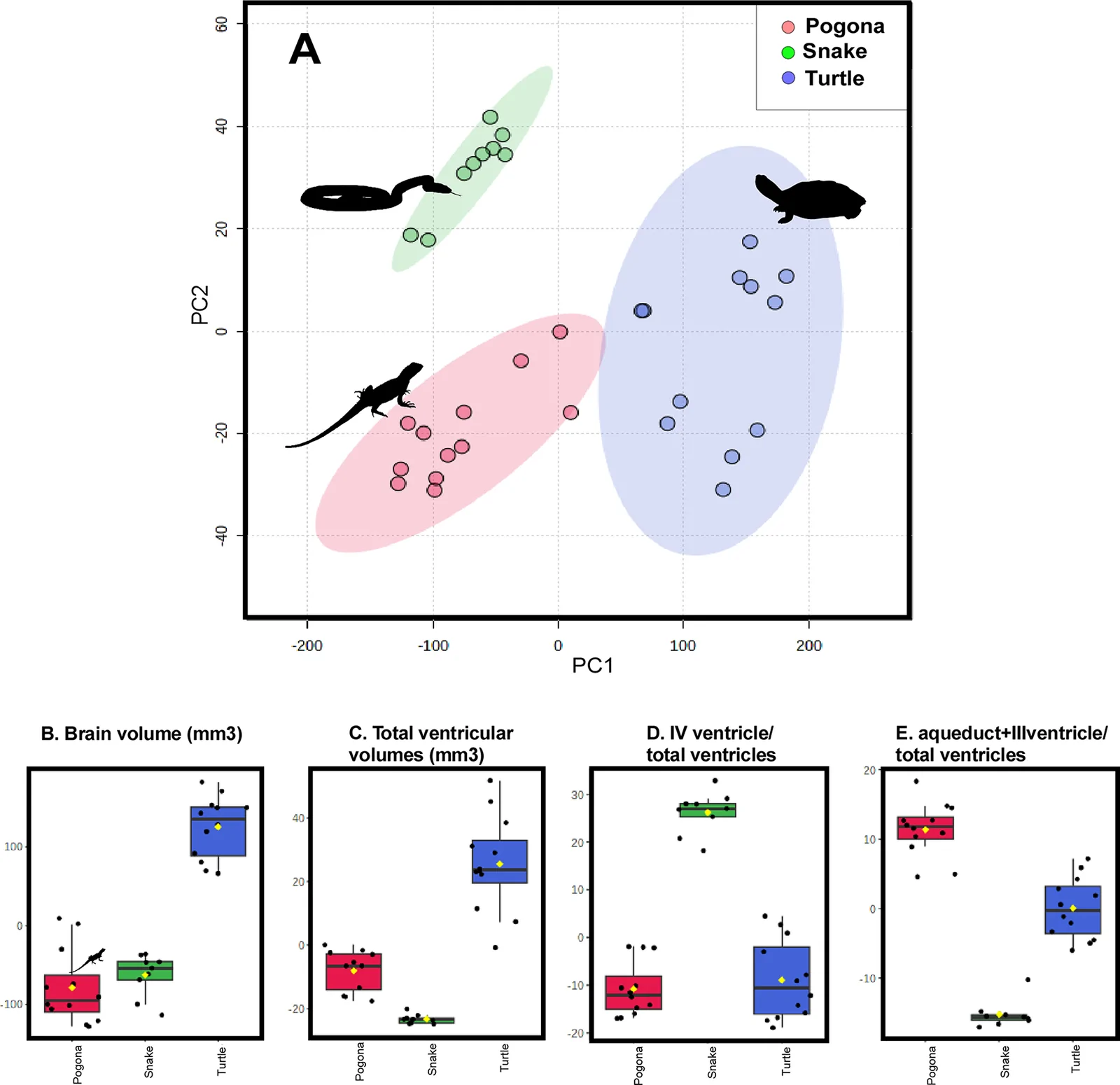
Result of the principal component (PC) analysis of the brain segmentation. Scores plot for the two PCs, PC1 vs. PC2 reveal separation of three species (**A**). Two Box-plot of the most significant variables: brain volume (**B**) is the most significant in PC1, and total ventricular volume (**C**) is in PC2. The concentrations in both variables were normalized and measured in mm^3^

Our results demonstrated that the three groups of our interest showed clear differences, in terms of brain and total ventricular volumes. Special attention should be paid to the turtles, which in all cases presented proper characteristics when compared to lizards (Pogona) and snakes (Fig. 9). Unsupervised Principal Component (PC) analysis was performed on the entire dataset. As a result, Fig. 9A shows the scores plot for the first two PCs (PC1 and PC2). The figure shows how the PC analysis allowed to correctly classify the three sauropsida species used in the present work. In the principal component plots, the three species are well separated. According to the graph, the first principal component (PC1) allows a clear differentiation between turtles and the other two species, while the second principal component (PC2) separates between turtles and pogonas, and shows differences between Pogonas and snakes.

In terms of volumetric analysis (Fig. 9B-E), the volume of the encephalon (Fig. 9B) together with the total volume of the ventricle (Fig. 9C) are the measures that allow a greater separation of turtles (blue) from pogonas (red) and snakes (green). While the fourth ventricle versus total ventricle volume (Fig. 9D) contribute to state a better separation of snakes from turtles and pogonas. The ratio of aqueduct + third ventricle versus total ventricle volume contributes to a better separation of the three species (Fig. 9E).

## Discussion

**Fig. 10:**
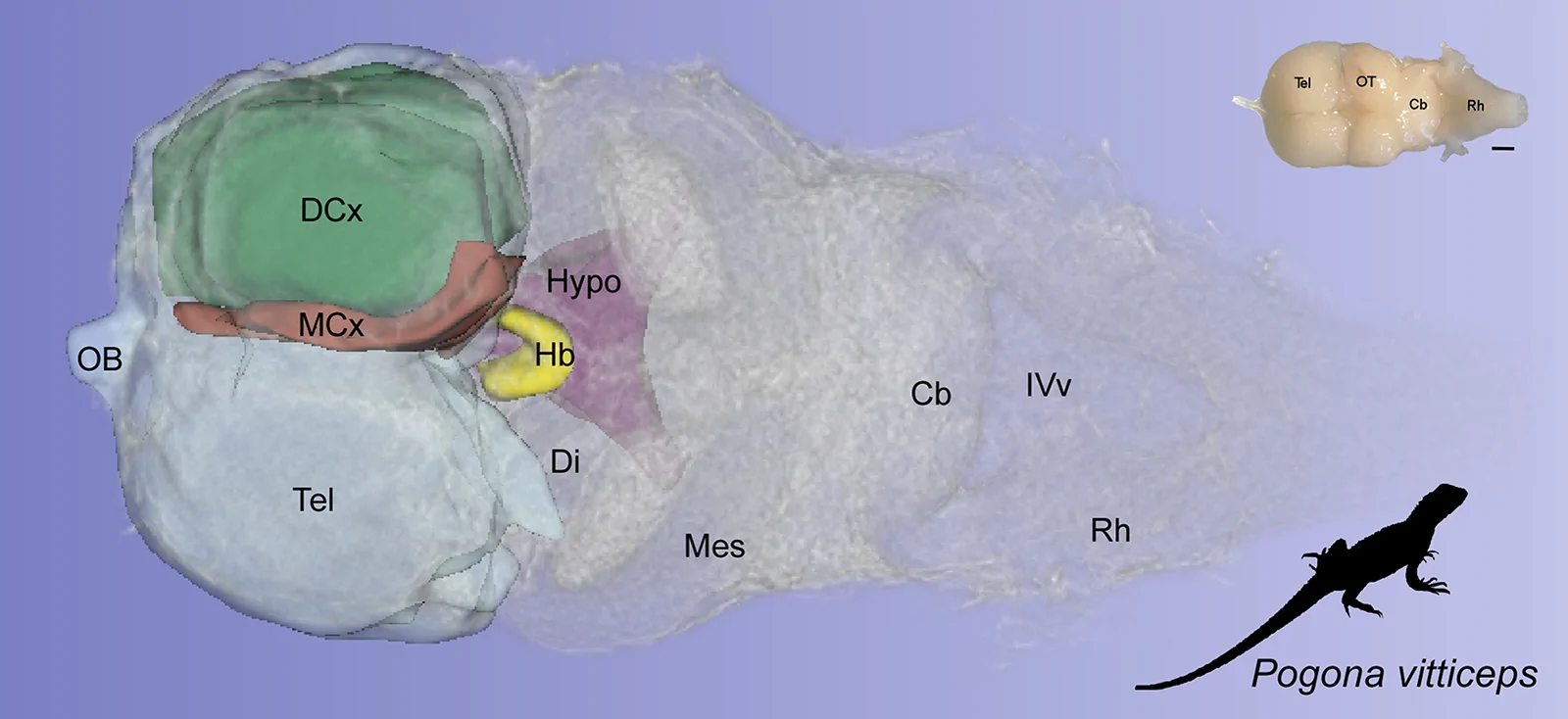
Three-dimensional reconstruction of specific brain regions in the brain of *Pogona vitticeps* showing in different colors the brain regions versus the total brain volume. In the upper right corner is shown the image of the dissected *Pogona vitticeps* brain (the same as in Fig. 2), with the scale bar, evidencing the coincidence in the reconstruction of the model. See abbreviations

Reptiles are the third major group of amniotes and in particular, with more than 10,000 species, squamate comprise the second largest group of terrestrial vertebrates (Pyron et al. 2013; Reeder et al. 2015; Uetz et al. 2022). For the past years, they have been considered as an ideal group for evolutionary brain studies (Striedter, 2005). In this context, in the last two decades, brain digital atlases based on MRI have been extended to non-humans species, including squamates (Kabli et al. 2006; Dorr et al. 2008; Poirier et al. 2008; Yopak and Frank 2009; Ullmann et al. 2010a; Ullmann et al. 2010b, 2012, 2013b, 2014b; Vellema et al. 2011; Simões et al. 2012; Hoops et al. 2017, 2018, 2021; Billings et al. 2020; Foss et al. 2022). In the present study, we have used the combination of MRI and histological analysis (DAPI) to map the forebrain (prosencephalon) of the turtle *Trachemys scripta*, the lizard *Pogona vitticeps* and the snake *Python regius* Fig. 10.

Our MRI analysis allowed the identification of three cortical structures in the telencephalon, tentatively identified by their anatomical position, not by other differences in organization, which is evident by DAPI-type nuclei staining, structure or, of course, function: dorsal, medial, and lateral. These cortical divisions have been also shown in other reptile species (Nieuwenhuys et al. 1998; Puelles 2017; Ulinski 1990), different to what happens in Hoop et al. (Hoops et al. 2017), who identify four cortical zones: the medial, dorsomedial, dorsal, and lateral. The cellular organization, specifically the layers observed by DAPI, was not identified in our MRI results. Nevertheless, our MRI results facilitate the anatomical regionalization of the cortex and the examination of size variations. The medial cortex was described by Hoops et al. (Hoops et al. 2017) as being different depending on the lizard group. In our case, by the use of DAPI, we demonstrate that the medial cortex showed similitudes and differences between the three species. A common characteristic was the presence of three distinct cell layers, with a central darker plate surrounded by two plexiform layers. In the case of the turtle, MRI demonstrated that the extension of the medial cortex was clearly greater in comparison to the other two. This fact supports the theory that this species is evolutionarily closer to crocodiles than to other reptiles (Green et al. 2014).

The dorsal cortex in reptiles has been previously described as a layered structure in an MRI atlas (Hoops et al. 2017). Our MRI analysis was successful in identifying the region of the dorsal cortex in turtles, snakes, and lizards, although we were not able to identify the layers. In sauropsidians, the lateral pallium includes the olfactory cortex, which is similar to the piriform cortex found in mammals and birds (Bruce 2007). However, recent proposals regarding the organization of the pallium suggest that, at least in lizards, it may also contain a ventropallial component (Desfilis et al. 2018). As in other reptiles (Butler and Hodos 2005; Billings et al. 2020), our study demonstrate that the lateral pallium extends along the lateral edge of the pallium, specifically along its entire rostro-caudal extent. In the dorsolateral region, it was visible in our MR images, and confirmed by means of DAPI, the pallial thickening, clearly seen in turtles [present results, in lacertid it could make up a small territory reduced only in rostral regions (Desfilis et al. 2018)]. The recent functional homology found between PT of turtles and aDVR of lizards (Norimoto et al. 2020) may explain this structural absence. It would be interesting to assess the functional implications of this structure and to be able to analyze its location and extent in different reptile species with different ecological and functional adaptations, as it would be easily analyzed by MRI. In addition, the dorsolateral amygdala has been described in this dorsolateral region in lizards and snakes (Lanuza et al. 1998; Lanuza and Halpern 1998; Hoops et al. 2018), anatomically identifiable in our models by MRI, although its embryological and/or anatomical relationship with the PT has not yet been analyzed. The dorsal ventricular ridge (DVR) was present in our three models of interest. A clear parceling of this region was not possible by MRI, however in this case, even a nuclei staining does not allow a clear differentiation of the subdivisions of this structure, as specific markers are needed. According to our results, the most remarkable anatomical region in the ventrocaudal pallium was the spherical nucleus, a structure that appeared as a layered formation in lizards and snakes but was missing in turtles. The layered organization of this structure is evident by MRI and by DAPI, in this case with cellular resolution. This nucleus has been described as being functionally involved in chemosensory processing, particularly vomeronasal, and thus especially developed in snakes and lizards with a forked tongue (Cooper 1997; Halpern and Kubie 1980; Schwenk 1993). Based on our analysis in the lizard *P.vitticeps*, although with a non-forked tongue, it showed a very clear spherical nucleus. This situation has also been described in the telencephalon of another agamid lizard by MRI (Hoops et al. 2018). Therefore, additional studies are needed to further investigate this finding and compare the size of the spherical nucleus in lizards and snakes. These investigations may provide valuable insights into the behavioral and ecological adaptations of these species, as the spherical nucleus may be associated with a unique and currently unknown pattern of olfactory connections related to the vomeronasal system.

In the subpallial region of these three models, the presence of highly conserved basal ganglia has been described [reviewed in (Marín et al. 1998)]. Using MRI, it is not possible to identify the specific nuclei composing this structure, in the same way that it is not possible through staining with DAPI, which allows cellular resolution, but not the establishment of boundaries when there are no cellular differences. However, this region is easily identified in all three models, likely due to the intensity of the fiber tracts, those of basal ganglia input/output. This opens the door to additional comparative analyses, as it could relate the anatomy and/or volume of this structure to motor characteristics of these animals. In this particular case, it is worth noting that snakes are limbless, and there are other limbless reptiles that may or may not have similarities in this region. It is the same in the case of the septum, where the region is clearly visualized by means of MRI, but specific nuclei cannot be identified, just as it was not possible with DAPI either. However, by MRI analysis it is possible to determine that it is relatively smaller in the pogona. Again, this interesting fact encourages to go deeper into comparative studies between species and the relative size of different structures in each of them.

The difficulty in distinguishing certain subdivisions of the hypothalamus has been noted in previous reports (Subhedar et al. 1989; Hoops et al. 2017; Billings et al. 2020; Ulinski 1986). In this case, it is worth noting that MRI was used to identify a specific region, while DAPI was used to identify cell types, but in both cases it is not possible to establish exact boundaries between nuclei. At this point it is important to keep in mind that nuclei can only be identified with specific markers. Our MRI results indicate the presence of large fiber tracts in turtles, lizards, and snakes. At the level of the diencephalic region, one of the great advantages of MRI was that, among other structures, the choroid plexus associated with this territory and the epiphysis were clearly identified. Considering that both structures are very sensible to the manipulation for histological processes, to have the possibility of observing their anatomical position in vivo is a great advantage. As pointed out in previous MRI reports on various reptile species, the identification of the prethalamic eminence in the diencephalon can be challenging. However, this was not our case, as we were able to recognize clearly the prethalamic eminence in turtles using MRI. (Simões et al. 2012; Lazcano et al. 2021). It was the same in the thalamic region, where both dorsal and ventral thalamic nuclei were readily outlined with MRI, which is in agreement with previous MRI atlases of the reptile brain [for example, (Hoops et al. 2017, 2021; Billings et al. 2020; Foss et al. 2022)].

According to our results, fiber tracts appear hypointense in MR images and are therefore highly identifiable. This is especially interesting in terms of comparative studies, as it allows to compare the size, location and characteristics of the tracts. Considering our three species of interest, turtles have the most identifiable tracts by MRI, to a lesser extent are the pogonas and finally the snakes.

Another interesting goal of the present study was to investigate the possible t (statistical) volume differences between turtles, lizards and snakes (these last two belong to the Order Squamata), being the turtles the model which showed bigger values in relation to brains and cerebral ventricles, which could be a sign of a potential correlation between brain size and evolutionary or phylogenetic encephalic adaptations. Taking into account that turtles and crocodilians are nested within the reptile tree, along with birds (Green et al. 2014; Hugall et al. 2007), and that crocodiles and birds have a higher degree of brain development in terms of brain size and/or certain abilities (Uetz et al. 2022; Billings et al. 2020), these results support the inclusion of turtles in Archelosauria (Crawford et al. 2015) and that it is possible to speculate that the larger brain size of turtles might also reflect a higher degree of development compared to Squamata. However, there is controversy between having a large brain, total number of neurons, brain volume and behavioral abilities, thus this comparative analysis by MRI and principal component analysis (PCA), rather than brain/body ratio or total number of neurons could be of additional interest. (Ngwenya et al. 2016; Dicke and Roth 2016; Olkowicz et al. 2016; Marhounová et al. 2019; Herculano-Houzel 2017) (Billings et al. 2020); Lazcano et al. 2021). We are aware that our volumetric analysis does not analyze neither the number of neurons of each species per area, nor the specific cognitive capacities. Consequently, it is risky to give a general statement about the brain of Squamates or Testudines, and the possible significance of having a larger brain. However, we consider that the analysis of the data by PCA is a powerful technique that allows us to identify which characteristics are the most influential in explaining comparatively the analyzed data, and we can also reduce their dimensionality, i.e. we could reduce the impact of brain or body size, which has already been discussed as not being good predictors of cognitive abilities and compare it, in future studies with the absolute number of neurons, data that are increasingly available in the literature. Thus, the present work is only an initial approximation and, therefore, at this point it is difficult to discuss special capacities or to postulate evolutionary rules, such as size versus ecological adaptations or size versus cognitive capacities, but it opens a door to the opportunity of this type of analysis including these experimental approximations. For this reason, further studies are needed to have a better understanding on the possible relation between volumetric brain differences and their possible relation to phylogeny or evolution.

## Concluding remarks

Although MRI is considered a more expensive technique than other imaging technologies, the development of low-field benchtop MRI machines with automatic self-adjustment of technical parameters has made it more accessible and affordable for veterinary clinics and preclinical research laboratories. The lower cost of this equipment, compared to traditional MRI machines, has also contributed to its more widespread use in veterinary medicine and research. MRI is a non-invasive technique, which makes it especially useful for wild or endangered species, and for those whose availability is limited, as could be the case of endangered or protected species. MRI facilitates inter- and intraspecific comparisons, assuming a breakthrough for evolutionary and comparative analysis [present results, (Ullmann et al. 2015a; Hoops et al. 2017, 2018; Billings et al. 2020; Nadkarni et al. 2019)]. In addition, it allows not only the comparison of the whole brain, but also the partial reconstruction of specific brain areas (Fig. 9). As we have shown, in most, if not all, brain regions, the level of detail offered by MR imaging is sufficient to tentatively identify specific structures and thus to perform partial three-dimensional reconstructions of areas. These can also be related to the whole brain, the ventricles, some regions versus others, etc. In addition, as shown in the present analysis, MRI-based studies can provide quantitative information, e.g., volumetric measurements for comparative neurobiological studies (Corfield et al. 2008; Oelschläger et al. 2008; Yopak and Frank 2009; Tullo et al. 2019). This opens a huge window of opportunity in anatomical studies in general and again in comparative studies. Because these data (including the data in this study) could be analyzed by comparing specific structures, such as the pallium vs. the subpallium, the pallium vs. the thalamus, or the olfactory bulb with the nucleus sphericus. And all these comparisons could be related to behavioral differences and/or ecological adaptations. This information is also a powerful tool in taxonomic or group analysis, since, as we have demonstrated in the present study, there are differences in terms of volume between the different orders analyzed that can be useful in taxonomic aspects. Given that MRI atlases may have a lower resolution compared to those generated from histological approaches, the combination of MRI and histology is a valuable opportunity to study the brain in greater depth (Garin et al. 2022).

## Electronic supplementary material

Below is the link to the electronic supplementary material.

Supplementary Material 1

## Data Availability

The authors confirm that the data supporting the findings of this study are available within the article (figures and supplementary figures). In addition, the raw data that support the findings of this study are available for viewing and downloading at the folder MRI DATA OF SAUROPSIDA MODELS: (https://drive.google.com/drive/folders/1QsQVL6S6oz_mRXavYt4LpCXtSKJgxbWZ?usp=sharing).

## Abbreviations

Acc: Nucleus accumbens
aLCx: anterior lateral cortex
BG: Basal ganglia
BST: Bed nucleus of the stria terminalis
BSTM: Bed nucleus of the stria terminalis medialis
Cb: Cerebellum
CH: Cortical hem
ChP: Choroid plexus
cp: Pallial commissure
DB: Diagonal band
DCx: Dorsal cortex
Di: Diencephalon
DLA: Dorsolateral amygdala
DVR: Dorsal ventricular ridge
Hb: Habenulae
Hypo: Hypothalamus
LCx: Lateral cortex
lfb: lateral forebrain bundle
Ma: Mammilary bodies
MCx: Medial cortex
MeA: Medial amygdala
Mes: Mesencephalon
NS: Nucleus sphericus
OB: Olfactory bulb
oc: Optic chiasm
OT: Optic tectum
ot: Optic tract
POA: Preoptic area
PT: Pallial thickening
PTh: Prethalamus
PThE: Prethalamic eminence
PV: Paraventricular hypothalamic region
Rh: Rhombencephalon
S: Septum
SPa: Subparaventricular zone of the hypothalamus
Str: Striatum
Tel: Telencephalon
Th: Thalamus
Tht: Thalamic hypothalamic tract
Tub: Tuberal area of the hypothalamus
v: ventricle
