# Correction: Comparative MRI analysis of the forebrain of three sauropsida models

**DOI:** 10.1007/s00429-026-03208-3

**Published:** 2026-10-25

**Authors:** S. Jiménez, I. Santos-Álvarez, E. Fernández-Valle, D. Castejón, P. Villa-Valverde, C. Rojo-Salvador, P. Pérez-Lloret, M. J. Ruiz-Fernández, S. Ariza-Pastrana, R. Martín-Orti, Juncal González-Soriano, Nerea Moreno

**Affiliations:** 1 Achucarro Basque Center for Neuroscience, Scientific Park of the University of the Basque Country (UPV/EHU), Leioa, Bilbao, 48940 Spain; 2 Departament Section of Anatomy and Embriology, Faculty of Veterinary, Complutense University, Avenida Puerta de Hierro s/n, Madrid, 28040 Spain; 3 ICTS Bioimagen Complutense, Complutense University, Paseo de Juan XXIII 1, Madrid, 28040 Spain; 4 Palmitos Park Canarias, Barranco de los Palmitos, s/n, Maspalomas, Las Palmas, 35109 Spain; 5 Department of Cell Biology, Faculty of Biological Sciences, Complutense University, Avenida José Antonio Nováis 12, Madrid, 28040 Spain

**Correction: Brain Structure and Function (2024) 229:1349–1364** https://doi.org/10.1007/s00429-024-02788-2

In this article, the co-author name has been incorrectly processed as “P. Pérez Llorens” but it should have been reads as “P. Pérez-Lloret”.

The original article has been corrected.

