# Supplementary material for: Comparative MRI analysis of the forebrain of three sauropsida models
Source: Brain Struct Funct. 2024 Mar 28;229(6):1349–64. doi: 10.1007/s00429-024-02788-2 (PMC11176103; doi:10.1007/s00429-024-02788-2)
Supplement: Supplementary file 1 — Supplementary Material 1 [file 429_2024_2788_MOESM1_ESM.docx]

supplementary data

(4 pages)

**Manuscript entitled: COMPARATIVE MRI ANALYSIS OF THE FOREBRAIN OF THREE SAUROPSIDA MODELS**

**Table of contents**

**1) Section S1. MRI - M&M of additional acquired experiments not shown in the text.**. .….p.2.

1. **Section S1.**  MRI - Material and Methods of additional experiments acquired during the experimental work not shown in the text.

Additional MRI experiments were acquired during the brain studies of the sauropsida species and are not shown in the main text. The different MRI experiments performed, as well as their main average acquisition parameters, are shown in this supplementary section. These experiments have been used to support the identification and segmentation of the structures.

The total MRI raw data (DICOM format) of the sauropsida models analyzed in the present study are available for viewing and downloading at the folder MRI DATA OF SAUROPSIDA MODELS.(<https://drive.google.com/drive/folders/1QsQVL6S6oz_mRXavYt4LpCXtSKJgxbWZ?usp=sharing>).

The additional MRI experiment performed were:

- SET1WI3D – **S**pin **E**cho **T1** coronal **W**eighted **I**mages **T**hree **D**imensional.

Three-dimensional T1WI were acquired using a rapid acquisition with relaxation enhancement (RARE) technique, with a repetition time (TR) = 214 s, echo train length = 2, effective echo time (TE) = 8 ms, number of averages = 6, field of view (FOV) = 32 × 32 × 16 mm^3^. The acquired matrix size was 250 × 90 × 70 (resolution 0.100 mm x 0.200 mm x 0.200 mm) and the total acquisition time ~ 180 minutes. This experiment was reconstructed in coronal and axial orientation with an isotropic resolution of 0.1 mm^3^.

- GET1T2*WI3D – **G**radient **E**cho **T1/T2*** coronal **W**eighted **I**mages **T**hree **D**imensional.

Tree-dimensional T1/T2*WI were acquired using a Fast Low Angle Shot (FLASH) MRI-sequence, with a TR = 56,8 ms, TE = 3.05 ms, flip angle = 15°, number of averaged experiments = 12, field of view (FOV) = 32 × 32 × 16 mm^3^. The acquired matrix size was 200 × 200 × 50 (resolution 0.160 mm x 0.160 mm x 0.320 mm) and the total acquisition time ~ 120 minutes. This experiment was reconstructed in coronal and axial orientation with an isotropic resolution of 0.1 mm^3^.

- GET1WI3D – **G**radient **E**cho **T1** coronal **W**eighted **I**mages **T**hree **D**imensional.

Tree-dimensional T1/T2*WI were acquired using a Inta Gate Fast Low Angle Shot (IG- FLASH) MRI-sequence, with a TR = 40.0 ms, TE = 12.0 ms, flip angle = 30°, number of

averaged experiments = 10, field of view (FOV) = 32 × 32 × 16 mm^3^. The acquired matrix size was 256 × 256 × 32 (resolution 0.125 mm x 0.125 mm x 0.500 mm) and the total acquisition time ~ 60 minutes. This experiment was reconstructed in coronal and axial orientation with an isotropic resolution of 0.1 mm^3^.

- SEDWI3D – **S**pin **E**cho **D**iffusion coronal **W**eighted **I**mages **T**hree **D**imensional.

Three-dimensional DWI were acquired using a rapid acquisition with relaxation enhancement (RARE) technique, with a TR = 2400 ms, echo train length = 4, effective echo time (TE) = 20 ms, number of averages = 4, field of view (FOV) = 32 × 20 × 16 mm^3^. The acquired matrix size was 180 × 100 × 40 (resolution 0.200 mm x 0.200 mm x 0.400 mm) and the total acquisition time ~ 180 minutes. This experiment was reconstructed in coronal and axial orientation with an isotropic resolution of 0.1 mm^3^.


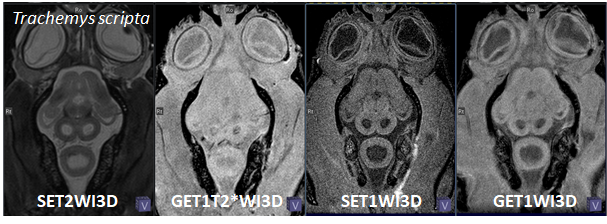

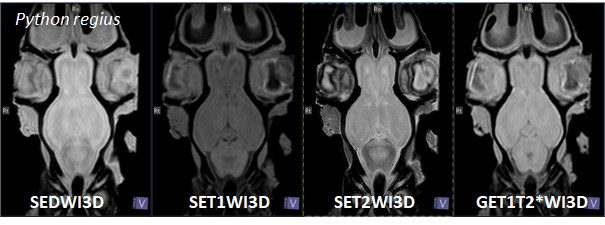


**Figure S1** shows different MRI contrast images in the *Trachemys scripta* and *Python regius* models.
